# The Introduction and Establishment of Four Invasive Insect Species in Serbia

**DOI:** 10.3390/insects14090728

**Published:** 2023-08-24

**Authors:** Mihaela Kavran, Aleksandra Konjević, Dušan Petrić, Aleksandra Ignjatović Ćupina

**Affiliations:** Center of Excellence, Faculty of Agriculture, University of Novi Sad, Trg Dositeja Obradovića 8, 21000 Novi Sad, Serbia; mihaela.kavran@polj.edu.rs (M.K.); dusanp@polj.uns.ac.rs (D.P.); cupinas@polj.uns.ac.rs (A.I.Ć.)

**Keywords:** urban zone, mosquitoes, mapping, mosquito-borne diseases, stink bugs, nuisance

## Abstract

**Simple Summary:**

Today, we are faced with urbanization and growing urban environments. Therefore, the number of insect species in cities is decreasing. However, urban insect pests, particularly populations of invasive species, are not limited by the factors that limit native insect populations. This article aims to describe four scenarios of the successful spread of invasive species in urban areas. Two mosquitoes and two stink bug species were selected for this study: the Asian tiger mosquito, the Japanese bush mosquito, the brown marmorated stink bug, and the southern vegetable stink bug. Regardless, mosquito species are considered insects of medical importance and stink bugs cause significant damage to agricultural production. What these four species have in common is their role as nuisance pests in urban environments.

**Abstract:**

Urban areas are often populated by specific species of insects, some colorful and appealing, such as ladybugs and butterflies, and others irritating as nuisance bitters or as vectors of pathogens of public health importance. Mosquitoes in urban areas often utilize habitats adjacent to human residences, while phytophagous insect species such as stink bugs often colonize ornamental plants and utilize human-made structures including houses as overwintering shelters. This article discusses the early detection and the current distribution of two invasive mosquito species, *Aedes albopictus* Skuse 1894 and *Ae. japonicus* (Theobald 1901), in Serbia, introduced in 2009 and 2018, respectively. From the first findings until today, regular monitoring has been carried out and the establishment of both species in the newly invaded areas has been confirmed. Both species can become nuisance species, especially at high population densities, but more importantly, they are capable of transmitting a wide variety of arboviruses of public health importance. This article also discusses two invasive stink bug species *Halyomorpha halys* Stål 1855 and *Nezara viridula* Linnaeus 1758, introduced in Serbia in 2015 and 2008, respectively. These two stink bug species have also been monitored, and the establishment of their populations in the country has been confirmed. Both species have caused damage to a wide range of crops and ornamental plants and sometimes become nuisance pests in urban areas.

## 1. Introduction

There are two primary ways that invasive insect species expand their ranges: (1) through migration, usually over short distances, and (2) through human activities, usually over long distances. Human activities such as global trade or travel can lead to the passive spread of insect species that were previously confined to their home ranges. Populations of species that can adapt to new ecosystems may expand rapidly, especially in the absence of natural enemies, and may become pests. This may lead to economic injury or vector-borne disease (VBD) outbreaks. 

Some mosquito species are of public health concern, especially when they occur in large population densities; they can be a biting nuisance but can also transmit pathogens of public importance, leading to vector-borne disease outbreaks. Collecting data on vectors of public health concern is critical to understanding the level of risk of vector-borne disease and determining the actions that need to be taken [1]. Invasive mosquito species are characterized by their ability to colonize new areas; they can displace or coexist with native mosquito species, but more importantly, they pose a public health and/or a veterinary threat [2]. Vector-borne diseases can significantly impact the socioeconomic development of some countries. Europe has a long history of both endemic and epidemic autochthonous transmission of vector-borne diseases. The main concern, however, is that the risk of vector-borne disease transmission in Europe is increasing because exotic vectors and pathogens are increasingly introduced through international travel and trade [1]. Some of these diseases are newly emerging or re-emerging after long absences; others are continuously spreading. The emergence or reemergence of VBDs is often associated with changes in ecosystems, human behavior, and climate. Although the number of autochthonous VBD infections in European countries is still relatively low, current predictions and models show an increasing trend [3].

Invasive mosquito species may remain undetected for long periods of time, as was the case with *Ae. japonicus* (Theobald) in Switzerland. The initial detection was after an areawide mosquito survey following numerous complaints from residents in an area of approximately 1400 km^2^. The detection of *Ae. japonicus* over such a wide area led to the conclusion that the species had gone undetected for several years. Similarly, the Asian tiger mosquito, *Ae*. (*Stegomyia*) *albopictus* Skuse, was present in Albania and Italy for 30 and 17 years, respectively, before the first outbreak of an *Ae. albopictus* transmitted disease was detected [3]. In contrast, in France, autochthonous cases of chikungunya and dengue were not detected until four years after *Ae. albopictus* had become established [4]. This suggests that favorable conditions that favor disease transmission by invasive mosquitoes are present in Europe [3]. These conditions are associated with pathogen introductions, the vectorial capacity of the established invasive mosquito populations, and the frequency of vector–host contacts. Climatic conditions can also have a direct impact on the pathogens, but also influence vector reproduction, activity, and survival [5,6]. Therefore, it is very important to monitor invasive mosquito species in each country to be prepared for possible VBD disease outbreaks and to establish appropriate action programs. 

Probably the invasive mosquito species of the greatest public health importance in Europe is *Ae. albopictus*. The public health importance of this species is reflected in its vector competence for a wide variety of arboviruses of public health importance and its wide distribution throughout almost all European countries [3]. 

Species that have attracted a great deal of attention in both urban and agricultural areas include stink bugs, which can seriously threaten agricultural production of many crops, fruits, and vegetables. These species overwinter in urban areas, invading houses, apartments, trains, buses, cars, and many other human-made structures. Sometimes, thousands of stink bugs can occupy a house and irritate the inhabitants of households. In the summer, stink bugs feed on a variety of plant hosts and devastate crop plantations, which greatly affects agricultural production. Two stink bug species from the family Pentatomidae, the brown marmorated stink bug, *Halyomorpha halys* Stål, and the green vegetable bug, *Nezara viridula* L., have been causing a great deal of damage to crop plantation across Europe. These two invasive species were first reported in urban areas and transportation hubs [7,8], from where they migrated to cultivated plants in the fields. 

## 2. The Asian Tiger Mosquito, *Aedes albopictus* Skuse (Diptera, Culicidae)

### 2.1. Distribution

*Aedes albopictus* originated in tropical forests of Southeast Asia, and it has spread to many parts of the world [2]. *Aedes albopictus* was first detected in Europe in 1979, and the very first detection was in Albania [9]. Although it was established in Albania, it was not reported in other parts of Albania or in any other European country until 1990, when it was detected in Italy [10]. Human activities, especially the used tires and the “lucky bamboo” trade [11,12,13,14], and the passive dispersal via public and private transportation have spread the species worldwide. This has resulted in a worldwide distribution of *Ae. albopictus*, and it is currently listed as one of the top 100 invasive species by the Invasive Species Specialist Group [15].

The successful invasion of *Ae. albopictus* is also the result of a combination of many abiotic and biotic factors, including ecological plasticity, strong competitiveness, globalization, inadequate mosquito surveillance, and inadequate mosquito control [12]. Climate change predictions suggest that *Ae. albopictus* will continue to successfully colonize areas beyond its current geographical range [3,13,14,16,17]. Strains of this species have adapted to temperate climates [3,12,13,14,15,16,17,18], and this may lead to further expansion of this species and possible disease transmission in many European countries.

### 2.2. Significance 

Asian tiger mosquito is an opportunistic feeder and feeds on a wide range of hosts [14]. It is characterized by its aggressive biting behavior, especially during the daytime hours [19]. The list of blood hosts includes humans, domestic and wild animals, reptiles, birds, and amphibians [20]; however, it prefers human blood [12]. It has the potential to become a serious health threat as a bridge vector of zoonotic pathogens to humans [21]. This mosquito species is a competent vector of chikungunya virus; dengue virus; 20 other arboviruses, including yellow fever virus, Rift Valley fever virus, Japanese encephalitis virus, West Nile virus, and Sindbis virus; as well as the nematode Dirofilaria, all of which are of public health importance to Europe [22,23]. Dengue virus transmission was detected in Serbia in 2015 [24]. A single human case was imported from Cuba, but fortunately, *Ae. albopictus* populations were not present in the vicinity of the patient and this possibly prevented a dengue outbreak. 

### 2.3. First Record in Serbia

The first detection of *Ae. albopictus* (Table 1) was reported on 1st September 2009 in an ovitrap at the Batrovci border crossing between Croatia and Serbia [25]. 

### 2.4. Monitoring

**Figure 1:**
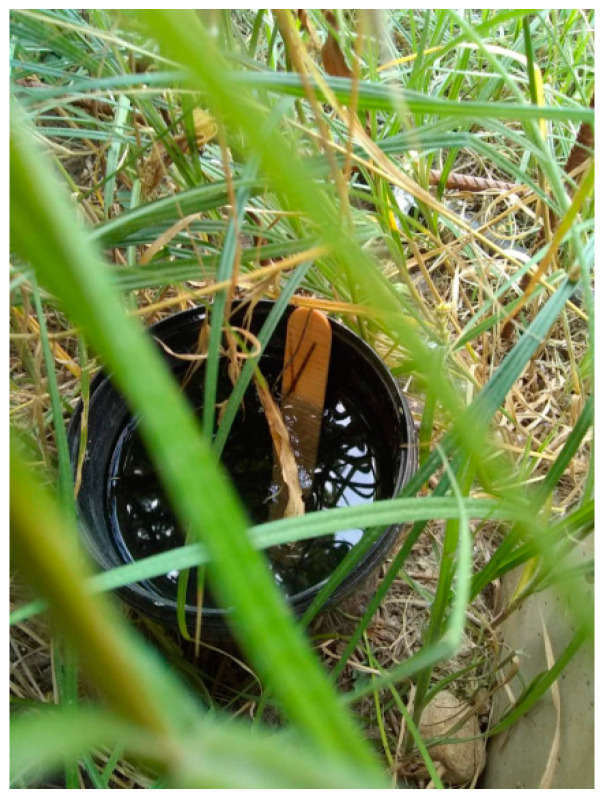
An ovitrap with a tongue depressor substrate used for monitoring of invasive *Aedes* mosquito species (Kavran, 2022).

Monitoring of the Asian tiger mosquitoes in Serbia started at the Batrovci border crossing in 2009. Since then, the monitoring zone has steadily expanded each year to include new locations. Monitoring is conducted by using standard ovitraps (Figure 1). The oviposition substrate is a wooden tongue-depressor. In 2022, monitoring was conducted at 40 sites in the city of Novi Sad. In the Vojvodina Province, 173 ovitraps were deployed covering the entire province. Sixteen more ovitraps were deployed in the Loznica municipality, Macva County.

**Figure 2:**
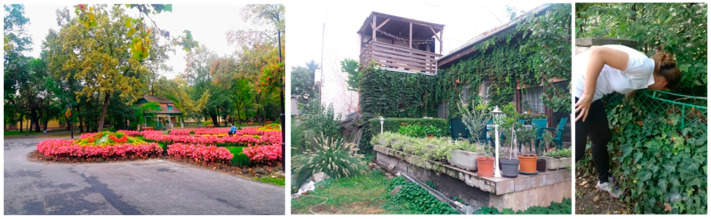
Ovitrap sites in Vojvodina (Kavran, 2022).

The exact locations were selected based on the biology and preferences of *Ae. albopictus*. Ovitraps were deployed into urban, suburban, and rural habitats. The ovitraps were placed in thick vegetation (Figure 2) close to human settlements. In addition, distribution information was obtained by passive monitoring, through photos submitted by the citizens via the Mosquito Alert app.

### 2.5. Distribution

**Figure 3:**
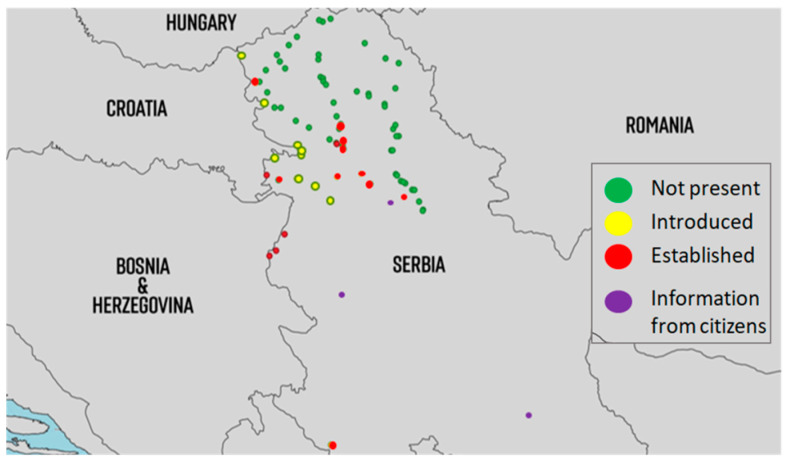
Distribution of populations of *Ae. albopictus* in Serbia from 2009 to 2022. (introduced = detected for the first time and afterwards only detected occasionally, mostly adult detections; established = multiple detections of all developmental stages throughout the season for at least two consecutive years at the same location; information from citizens were sent using email or Mosquito Alert app via high-quality photos).

From the first detection up to now, populations of *Ae. albopictus* have been present at the Batrovci border crossing as well as at sites along the highway from Batrovci to Ruma. In addition, *Ae. albopictus* has been present in Gostun, at the border with Montenegro, since 2011. It is assumed that this species was not established in Serbia until 2017, but it has been detected at the border crossings every summer during the peak travel period. In the first half of 2017, eggs of this species were detected in Batrovci at the beginning of the travelling season, suggesting that *Ae. albopictus* populations had been established in Serbia since 2017 (Petrić, unpublished data). The third location where this species is established is the urban area of Novi Sad, where populations of the Asian tiger mosquito have been continuously detected in several locations since 2018. Currently, *Ae. albopictus* has successfully spread to all parts of Novi Sad. Populations of *Ae. albopictus* have also been reported in many other areas, such as Inđija, Apatin, Kuzmin, Ruma, Sremska Mitrovica, Belgrade, Loznica, Niš, and Valjevo (Figure 3). All border crossings with Croatia were also positive for populations of the Asian tiger mosquito. The invasion of Serbia by the Asian tiger mosquito has been very successful.

### 2.6. Applied Control Measures

The control of *Ae. albopictus* is very difficult and complex due to the variety of larval sites, catch basins, types of water containers, or artificial breeding sites that could be inhabited by this invasive species. Traditionally, invasive mosquito control has been performed via adulticide and larvicide treatments at the provincial or municipality level. However, conventional mosquito control measures alone have a very low level of success against *Ae. albopictus* populations. The best approach is the integrated mosquito

Management approach (IMM): One of the promising methods is the sterile insect technique; it was carried out for the first time in Serbia in 2022 (Petrić et al., unpublished data).

## 3. Japanese Bush Mosquito, *Aedes japonicus* (Theobald) (Diptera, Culicidae)

### 3.1. Distribution

The Japanese bush mosquito is an Asian species native to Japan, Korea, southern China, Taiwan, and the Russian Federation [26]. Its first finding in Europe was reported in 2000 from northwestern France [22]. Subsequently, *Ae. japonicus* was found in Belgium in 2002 [27], in Switzerland and Germany in 2008, in Austria and Slovenia in 2011, and in the Netherlands and Hungary in 2012 [28,29,30]. In 2013, it was reported in Croatia [31]. Its spread continued in Liechtenstein and Italy in 2015; in Bosnia and Herzegovina in 2017; and Serbia, Spain, and Luxembourg in 2018 [31,32,33,34,35,36]. The most recent reports of its introduction (2020) are from Romania [37] and the Czech Republic in 2021 [38]. In Europe, *Ae. japonicus* became established in Belgium and was successively detected in Switzerland and Germany, where it spread rapidly [28,39]. 

*Aedes japonicus* has less specific aquatic habitat requirements compared with *Ae*. *albopictus*, which may facilitate the further spread of this species [22]. In its native range, *Ae. japonicus* colonizes habitats with tree cavities, but this has not often been reported for newly established areas in Europe [40]. Unlike native European mosquito species of the genus *Aedes*, this species has a relatively short flight range [41]. *Aedes japonicus* prefers forested habitats and colonizes rock pools and tree holes as natural breeding sites, as well as artificial water recipients: containers, used tires, birdbaths, toys, vinyl tarpaulins used to cover swimming pools, catch basins, rainwater tanks, stone vessels, drinking fountains, tin cans, bath tubs, roof gutters, flower vases, plant pots, street gutters, rain barrels, buckets, pans, etc. [42]. 

### 3.2. Significance 

This species is also known as the Asian bush mosquito, a very aggressive daily biter. It prefers to feed on mammals and humans [42,43,44] but can also use blood from other hosts such as birds, rodents, etc. [45,46,47]. Adults are commonly found in forested areas [42] and are active during the day, dusk, and dawn [19]. *Aedes japonicus* prefers to bite humans outdoors and occasionally in houses [22]. This species has tested positive for West Nile virus in many cases in the United States [19,42]. Laboratory experiments have shown that the Japanese bush mosquito is a competent vector of West Nile virus [48]. Laboratory studies have also confirmed that *Ae. japonicus* is a competent vector of Japanese encephalitis virus [49] and La Crosse virus [46], and a moderately effective vector of Saint Louis encephalitis virus [47], Eastern equine encephalitis virus [45], Chikungunya virus and Dengue virus [50], and Rift Valley fever [51].

### 3.3. First Record in Serbia

The Japanese bush mosquito invaded Serbia in 2018 (Table 1). The species was first detected at the Ljuba border crossing with Croatia [36]. 

### 3.4. Monitoring

*Aedes japonicus* was monitored using the same type of ovitraps and passive monitoring as described above for the monitoring of Asian tiger mosquito. These two species were monitored simultaneously using the same traps. 

### 3.5. Distribution

**Figure 4:**
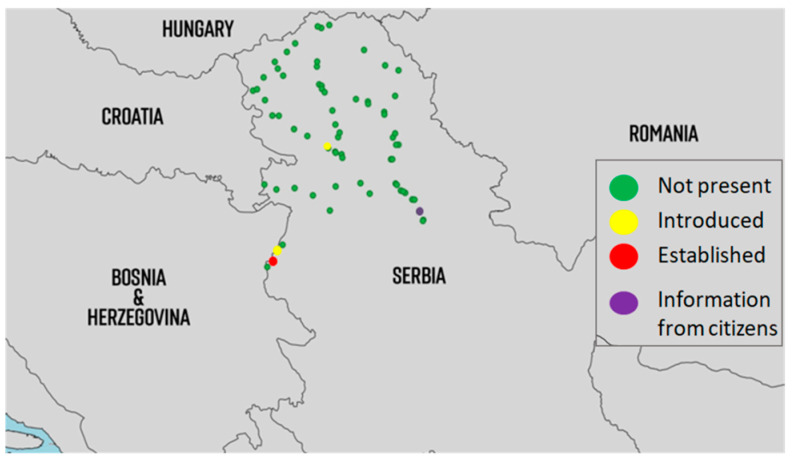
Distribution of populations of *Ae. japonicus* in Serbia from 2018 to 2022. (introduced = detected for the first time and afterwards only detected occasionally, mostly adult detections; established = multiple detections of all developmental stages throughout the season for at least two consecutive years at the same location; information from citizens were sent using email or Mosquito Alert app via high-quality photos).

In 2022, this species was detected in four locations (Figure 4). Its occurrence was detected in Novi Sad (semi-urban environment) and in the Loznica municipality in two places around the city (one urban and one semi-urban). Passive monitoring showed that the species occurred in Opovo (South Banat County). Although ovitraps were placed in Opovo, we have not detected *Ae. japonicus* there so far. A possible explanation for this is that, in monitoring, we focused more on the biology and preferences of *Ae. albopictus* in our studies and therefore neglected the preferences of the other mosquito species. Based on the report we obtained through passive monitoring, we believe that the population of *Ae. japonicus* in Serbia may be underestimated. Future efforts to monitor the distribution of *Ae. japonicus* should be improved by focusing on their preferences. 

### 3.6. Applied Control Measures

Fortunately, this species is not yet widely distributed in our country. Considering the similar biology of these two invasive mosquito species (especially breeding sites, method of introduction, and preferences for mammals), the approach to their prevention and control should consist of a similar strategy. In Serbia, there are no measures specifically focused on controlling the population of *Ae. japonicus*. The control of *Ae. japonicus* is part of regular mosquito control campaigns in the country.

## 4. Brown Marmorated Stink Bug, *Halyomorpha halys* Stål (Hemiptera, Pentatomidae)

### 4.1. Distribution

The brown marmorated stink bug (BMSB) is a species native to East Asia [52] that successfully established its populations outside this region, first in the USA in the late 1990s [53] and later in Europe, in 2004 from Liechtenstein and Switzerland [7,54,55]. Soon after these finds, the species spread and became established in almost all European countries: Germany [56], France [57], Italy [58], Hungary [59], Austria [60], Romania [61], Greece [62], Spain [63], Bulgaria [64], Georgia and Abkhazia [65], Russia [66,67], Slovakia [68], Slovenia [69], Croatia [70], Bosnia and Herzegovina [71], Albania [72], and the Republic of North Macedonia [8].

The most common route of spread of *H. halys* is transportation and trade of goods, which has been confirmed in many European countries [7,8,73]. The spread and increase in the BMSB population were first observed in large cities along the frequently used highways. However, their dispersal into surrounding agricultural areas most likely occurs though their active flight, with summer generations flying farther than overwintering adults [74]. The climate suitability model CLIMEX [75] has shown that climatic conditions in Europe are suitable and consistent for further spread of BMSM and that this species will most likely become economically important in almost all inhabited areas.

### 4.2. Significance 

The brown marmorated stink bug is an extremely polyphagous species that feeds on more than 106 ornamental plants in its native range [76,77]. Many vegetable, field, and even medicinal plants are on the list of endangered plant species [77], with the greatest economic losses occurring on plants of the Fabaceae and Rosaceae families. Its harmfulness is reflected in the fact that it feeds on a wide range of hosts, including crops, weeds, and ornamentals, providing it with a constant food source and opportunities to maintain populations. In Japan, the species mainly attacks fruits and soybeans, and in China, the species mainly attacks apples [78]. In the United States, it is harmful to apples, peaches, tomatoes, peppers, squash, cucumbers, sweet corn, soybeans, and beans [79]. Economically important damage to bell pepper plants was observed in Switzerland, in 2012 [80], and major damage to pears and other fruits was reported in Italy [81]. In Serbia, damage was observed on hazelnuts, soybeans, cherries, apricots, nectarines, apples, and blueberries. Infested fruits are deformed and have white spots, which soon develop into necrotic spots, leading to a rapid decline in the commercial value of the fruit and often resulting in complete destruction. Ornamental plants, which are usually excluded from any type of control program, serve as host plants where population growth is usually undisturbed and from which specimens spread and infest crops. The main urban hosts are Catalpa and newly planted Paulownia trees, which serve as hosts for both adults and nymphs.

**Figure 5:**
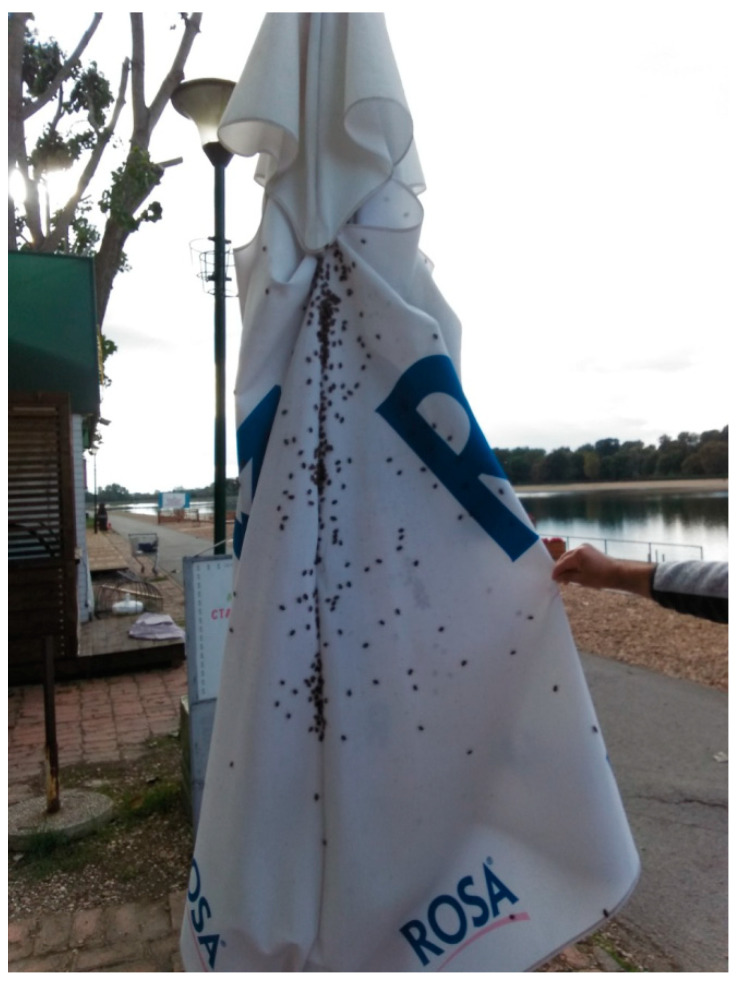
Adult *H. halys*, ready to overwinter in the urban area (Konjević, 2019).

In addition, this species is important in urban areas, where adults overwinter in protected areas (shelters) under the bark of woody plants (especially sycamore trees, Konjević, unpublished data), but also in houses, apartments, and other buildings in urban and semi-urban areas, where they escape the adverse effects of low temperatures and frost. Adults are sometimes found in large numbers in weekend houses, restaurants in green areas, and areas near forests (Figure 5). With its unpleasant odor released when the specimens are disturbed, the species become very unwelcome guest soon after the appearance. 

### 4.3. First Record in Serbia

*Halyomorpha halys* was detected in Serbia through a photo published on social media. Photo was made at the Serbian–Romanian border, and shortly after, adult specimens were collected in two cities, Vršac and Belgrade, in October 2015 [82]. Subsequently, a rapid spread of the population was observed, and the number of host plants increased from urban plants to agricultural crops [67]. The year of the first detection and establishment was given in Table 1. 

### 4.4. Monitoring

**Figure 6:**
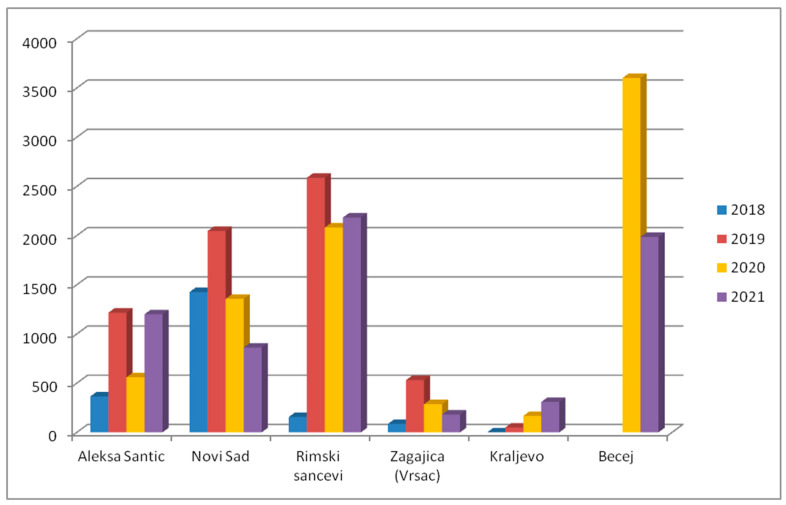
Total number of *H. halys* specimens collected with five dead-in traps during four consecutive years.

Monitoring of BMSB was initiated in 2016 and was conducted via pyramid dead-in traps (AgBio Company, Lodi, CA, USA) using aggregation pheromones (Tréce lures, Adair, OK, USA) placed in both urban and agricultural areas. The number of traps per country was increased each year and 42 traps were set in 2022. Traps were checked weekly from May to the end of September each year. Since the beginning of trapping, five traps were placed in the same location. The results showed the continuous spread of the species throughout the country and also the expansion of the population at most trap sites (Figure 6). 

### 4.5. Distribution

**Figure 7:**
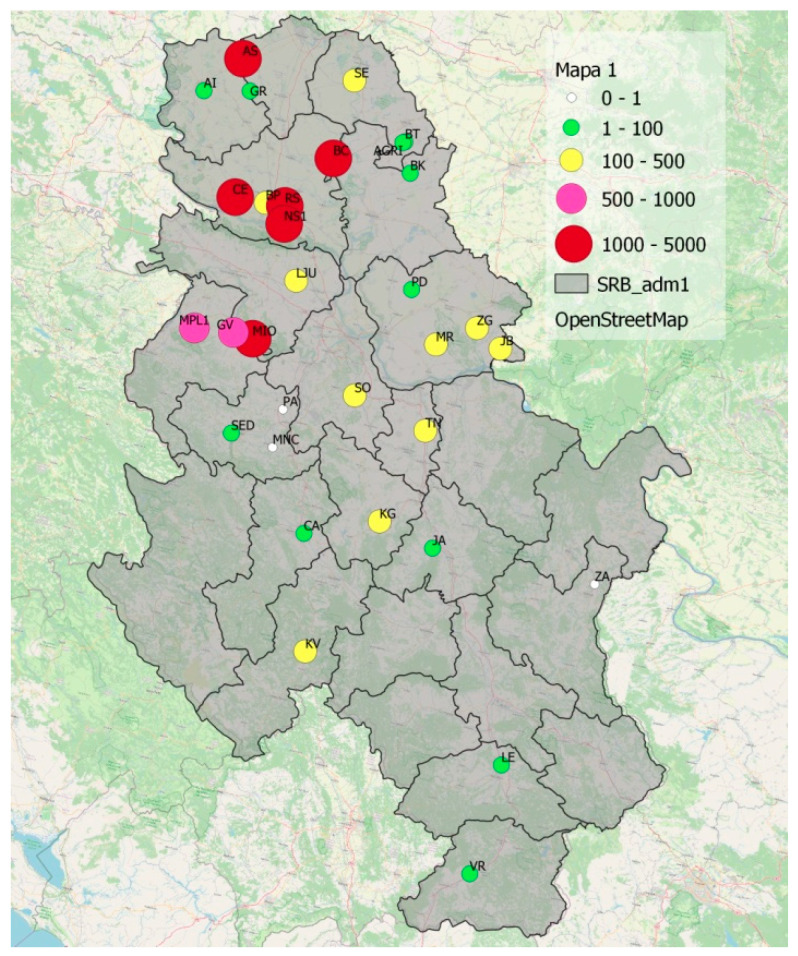
Abundance of the brown marmorated stink bug (*H. halys*) per region in 2021.

Today, BMSB is considered a well-established species in Serbia with increasing economic impact on agricultural production. In 2021, a total of 14,361 specimens were collected with traps, which was more than the total number of 11,214 specimens in the previous year. The distribution of specimens is not uniform across the country (Figure 7) due to the different distribution of favorable host plants and different environmental conditions (e.g., altitude, precipitation, and solar radiation). 

### 4.6. Applied Control Measures

The methods of controlling BMSB in Serbia are still based on the application of broad-spectrum insecticides, mainly a range of pyrethroids, carbamates, and neonicotinoids, which are usually not sufficiently effective. Chemical treatments are applied by farmers, who usually respond with increased insecticide applications. The main problem with control is the inconsistent approach by individual farmers, which results in specimens moving from untreated to treated fields, requiring new treatments. In urban areas, there is no treatment. Current research is focused on native or alien natural enemies on the territory of Serbia that could be effective in biological control.

## 5. Southern Green Stink Bug, *Nezara viridula* (Linnaeus), (Hemiptera, Pentatomidae)

### 5.1. Distribution

This species of uncertain geographic origin, most likely originating from East Africa and the Mediterranean [68], is currently distributed in tropical and subtropical regions of Africa, Eurasia, North and South America, and Australia [68]. Some authors suggest that the species has spread through human activities [83]. However, adults of *N. viridula* are strong fliers and their dispersal is usually rapid. The species exhibits a marked polymorphism. Freeman [84] described four different color variants, and Yukava and Kiritani listed five more [85,86]. According to later studies, polymorphism is defined as a necessity for dispersal and survival. A cryptic green body in summer and a brown one before winter are a good camouflage strategy. 

### 5.2. Significance

It is also a very nuisance species whose adults can be numerous in urban areas and can disturb people in resting and residential areas during the winter diapause. Similar to the BMSB, it emits an unpleasant odor when disturbed and can be numerous in homes. In addition to this nuisance factor, the species is a pest of many horticultural and field crops, including more than 30 plant families [87].

Adults and nymphs feed by sucking plant juices, concentrating primarily on fruits that are deformed and have low market value. A wide range of insecticides are used to control *N. viridula* and *H. halys*, many of which are harmful to beneficial insects and are restricted for use in residential areas.

### 5.3. First Record in Serbia

The southern green stink bug, also known as the green vegetable stink bug in Serbia, was first detected in Novi Sad and surrounding areas in 2008 and 2009 [88,89]. Years of the detection and establishment are given in Table 1. There is no evidence of the route by which it arrived in Serbia, except for the assumption that it was introduced by transport, similar to the BMSB. The first mass occurrence of this species was recorded in September 2011 in several locations around Novi Sad and Sombor [89]. Since then, it has spread throughout the country and is now considered one of the most common and numerous species in vegetable crops.

### 5.4. Monitoring

So far, *N. viridula* has not been systematically monitored in Serbia. The species is monitored only via visual inspection of its occurrence in different crops and urban areas. It is frequently found in dead-in traps together with BMSB, but we cannot rely on this number, due to the species-specific pheromone in the mentioned traps. The two species are found in similar locations, suggesting similar biology and overwintering preference. 

### 5.5. Distribution

As mentioned above, *N. viridula* is present and established throughout the country, increasing in regions where more vegetables are grown, such as in the northern part of Serbia, Vojvodina, but also in the southern parts, Leskovac and Vranje municipalities, where peppers and tomatoes are the dominant crops. Hidden dark places, where *H. halys* is abundant, also serve as good shelters for *N. viridula*. Garden plots and farms, as well as green areas with favorable ornamental plants, are the main reservoirs for the specimens, which spread uncontrollably and abundantly in such areas under suitable conditions. Spread to larger arable areas, where they cause considerable damage, is inevitable under these conditions.

### 5.6. Applied Control Measures

As mentioned earlier in BMSB, control of *N. viridula* also relies primarily on foliar applications of broad-spectrum synthetic chemicals to reduce pest damage. Because insecticide use has a negative effect on non-target organisms, it should be based on monitoring data, which are generally ignored. Therefore, farmers still lack effective methods to control *N. viridula*. The current research focus is on natural enemies and their effectiveness.

## 6. Conclusions

Long-term monitoring of mosquitoes and stink bugs in Serbia resulted in significant findings of four invasive alien species. Two mosquito species, *Ae. albopicus* and *Ae. japonicus*, are now considered established in certain areas of Serbia, while the stink bugs *H. halys* and *N. viridula* have established populations throughout the country, in both urban and agricultural habitats. 

The four invasive insect species have found their way to invade the countries of Central Europe, including Serbia, probably though different transport routs. Serbian highways connecting Central and Southern Europe are intensively used by cars and trucks throughout the year, especially in summer. Motor vehicles are the most suitable way for stowaway insects, such as the described stink bug species, to travel and expand their habitat over long distances [7,14,73]. This is also confirmed in Serbia by the fact that populations of *Ae. albopicus* were recorded in a larger number of urban and suburban habitats, while the Japanese mosquito, *Ae. japonicus* was detected in four locations, mostly near the border crossing. The first finding of *H. halys* also refers to the border crossing with Romania and a simultaneous finding in the urban part of Belgrade, 100 km away [82]. Only the possible routes of spread of *N. viridula* are unclear because the first mass occurrence was already recorded in soybean fields and many orchards [89], and then, the mass occurrence in urban areas followed.

The observed species that had previously established in surrounding countries found favorable conditions, in terms of climatic conditions and food, to establish their own populations, as mentioned above for each species (Figure 3, Figure 4 and Figure 7). Today, *Ae. albopictus* is considered the most widespread invasive mosquito species in Europe [90]. In addition to the transport sector, which is most responsible for the spread of this species, the high adaptability of this species has allowed it to successfully colonize new areas. In addition to passive dispersal through the Dracaena trade (lucky bamboo, *Dracaena sanderiana*), the trade in scrap tires has also contributed to the successful invasion [12]. The Asian tiger mosquito is on the EU list of the top 100 invasive species in the world [90]. Changes in land use, particularly urbanization and global warming, could further increase the competitive advantage of *Ae. albopictus* and *Ae. japonicus* over native mosquitoes through the use of artificial container habitats and further establishment in new areas [91]. Winter temperatures and annual average temperatures appear to be the most important limiting factors for the spread of invasive mosquitoes in Europe [92]. *Aedes albopictus* exists in tropical and temperate forms. The temperate form is known to establish in areas above 40 degrees north latitude. At this stage, when populations are widespread in Serbia, it is almost impossible to think of their eradication. However, all effective measures offered by integrated mosquito management must be considered to suppress and keep mosquito populations low to prevent future outbreaks.

Although some of the invasive species, such as *N. viridula*, have been present for more than a decade, control measures in Serbia to date have been based mainly on the use of chemical insecticides, which are sometimes ineffective. In almost all countries, stink bug control measures generally rely on insecticides, many of which affect natural enemies and parasites that may be present, which is a limitation for effective control strategies [81].

A promising management tool could be the use of an alien parasitoid species that would reduce stink bug populations not only in agricultural areas but also in urban areas. A good example is Italy, where two egg parasitoids were discovered and tested under natural conditions, *Trissolcus japonicus* (Ashmead) and *T. mitsukurii* (Hymenoptera: Scelionidae) [93,94]. Control measures in urban areas where stink bugs disturb people due to their numbers, unpleasant odors, and disoriented flying are very often neglected or difficult to implement. Therefore, natural enemies should be considered and used as a promising tool to control these agricultural and urban pests.

## Figures and Tables

**Table 1:** List of four monitored species introduced to Serbia with years of introduction and year of establishment or mass occurrence in case of the *N. viridula*.

Species	Year of Introduction	Year of Establishment/Mass Occurrence
*Aedes albopictus*	2009	2017
*Ae. japonicus*	2018	2022
*Halyomorpha halys*	2015	2017
*Nezara viridula*	2008	2010

## Data Availability

The data presented in this study are available in the article.
